# MMPs Regulate both Development and Immunity in the *Tribolium* Model Insect

**DOI:** 10.1371/journal.pone.0004751

**Published:** 2009-03-09

**Authors:** Eileen Knorr, Henrike Schmidtberg, Andreas Vilcinskas, Boran Altincicek

**Affiliations:** Interdisciplinary Research Center, Institute of Phytopathology and Applied Zoology, Justus-Liebig-University of Giessen, Giessen, Germany; Massachusetts General Hospital, United States of America

## Abstract

**Background:**

Matrix metalloproteinases (MMPs) are evolutionarily conserved and multifunctional effector molecules in development and homeostasis. In spite of previous, intensive investigation *in vitro* and in cell culture, their pleiotrophic functions *in vivo* are still not well understood.

**Methodology/Principal Findings:**

We show that the genetically amenable beetle *Tribolium castaneum* represents a feasible model organism to explore MMP functions *in vivo*. We silenced expression of three insect-type *Tribolium* MMP paralogs and their physiological inhibitors, TIMP and RECK, by dsRNA-mediated genetic interference (RNAi). Knock-down of MMP-1 arrested development during pupal morphogenesis giving phenotypes with altered antennae, compound eyes, wings, legs, and head. Parental RNAi-mediated knock-down of MMP-1 or MMP-2 resulted in larvae with non-lethal tracheal defects and with abnormal intestines, respectively, implicating additional roles of MMPs during beetle embryogenesis. This is different to findings from the fruit fly *Drosophila melanogaster*, in which MMPs have a negligible role in embryogenesis. Confirming pleiotrophic roles of MMPs our results also revealed that MMPs are required for proper insect innate immunity because systemic knock-down of *Tribolium* MMP-1 resulted in significantly higher susceptibility to the entomopathogenic fungus *Beauveria bassiana*. Moreover, mRNA levels of MMP-1, TIMP, and RECK, and also MMP enzymatic activity were significantly elevated in immune-competent hemocytes upon stimulation. To confirm collagenolytic activity of *Tribolium* MMP-1 we produced and purified recombinant enzyme and determined a similar collagen IV degrading activity as observed for the most related human MMP, MMP-19.

**Conclusions/Significance:**

This is the first study, to our knowledge, investigating the *in vivo* role of virtually all insect MMP paralogs along with their inhibitors TIMP and RECK in both insect development and immunity. Our results from the *Tribolium* model insect indicate that MMPs regulate tracheal and gut development during beetle embryogenesis, pupal morphogenesis, and innate immune defense reactions thereby revealing the evolutionarily conserved roles of MMPs.

## Introduction

Matrix metalloproteinases (MMPs) are evolutionarily conserved but differ in gene family member number in animal species ranging from Cnidaria to Vertebrata. Humans have, for example, 22 different homologs [1] whereas the fruit fly *Drosophila melanogaster* has only two homologs [2]. Each of the vertebrate MMPs has distinct but overlapping functions and substrate specificities, and together they can cleave numerous extracellular substrates, including virtually all extracellular matrix proteins [3]. They are involved in mammalian physiological processes such as development, wound healing, and inflammation as well as in pathological states including cancers, rheumatism and osteoarthritis [1]–[6]. Confirming their importance MMPs are tightly regulated at transcriptional and posttranslational levels. Proteolysis, allosteric interactions, and oxidative modification have been demonstrated to control MMP activities in the microenvironment of cells and tissues [7]–[8]. Moreover, specific physiological MMP-inhibitors have been identified including tissue inhibitor of metalloproteinases (TIMP) and reversion-inducing-cysteine-rich protein with kazal motifs (RECK). TIMPs have conserved cysteine residues forming disulfide bridges [9]–[11] and besides inhibiting MMPs, mammalian TIMPs have also been implicated in direct regulation of cell growth and apoptosis by interacting with the cell surface receptors integrin α3β1 and CD63 [11]. RECK encodes a glycosylphosphatidylinositol (GPI)-anchored glycoprotein containing multiple serine protease inhibitor-like motifs that negatively regulates MMPs *in vivo* [12], [13].

As their name implies, MMPs were originally described as proteinases capable of degrading extracellular matrix proteins such as collagens, elastin, proteoglycans, and laminins. However, recent studies have further revealed that MMPs exhibit pleiotrophic roles by both activating or inactivating other proteinases, proteinase inhibitors, clotting factors, chemotactic molecules, latent growth factors, growth factor–binding proteins, antimicrobial peptides, and cell-surface receptors [2], [7], [8]. They are multifunctional effectors with dual promoting and inhibiting activities on numerous physiological processes such as tissue growth and survival, angiogenesis, cell invasion, epithelial-to-mesenchymal transition, and immune surveillance [14].

In spite of their intensive investigation *in vitro* and in cell culture, physiological *in vivo* roles of MMPs are not well elucidated yet. Hence, to unravel specific underlying molecular mechanisms more research on feasible animal model organisms are urgently needed. For example, pharmacological inhibitors of MMP activity have been shown to profoundly affect development and homeostasis in species ranging from cnidarians to mammals [15], [16]. In mice, knock-out mutants indicate a redundancy among MMPs and only few MMPs have been found to be required specifically during bone and vascular remodeling and mammary development [2], [6]. Zebrafish lacking specific MMPs develop severe morphological abnormalities during embryogenesis including abnormal somitogenesis, organ development, and tissue architecture and are not viable [17]–[19]. In the fruit fly *D. melanogaster*, two MMPs have been identified [20], [21], which control larval tracheal growth and events of pupal morphogenesis [22]. This pioneering study generated for the first time mutant organisms which were completely depleted for MMP activity and provided deeper understanding of *in vivo* roles of individual MMP in the fly development. Additionally, a recent study indicated that both *Drosophila* MMPs modulate the responses of embryonic motor axons of defined neuronal populations to specific guidance cues [23], indicating suitability of insect model organism to elucidate novel MMP functions.

To better understand specific *in vivo* functions of MMPs, here we study their roles in the red flour beetle *Tribolium castaneum*. *Tribolium* has emerged as feasible model organism in insect evolutionary and developmental research because its complete genomic sequence is available [24] and it exhibits a more ancestral development when compared to *Drosophila* [25]. In this study, we identified orthologs of the two MMPs known from *Drosophila*, as well as a third MMP homolog, which we named MMP-3. This MMP-3 is present in *Tribolium* and, for example, in *Anopheles* but is absent in *Drosophila*, suggesting it may have been lost in *Drosophila*. In addition, we identified ten probably virus-derived MMPs in *Tribolium* with yet unknown functions. Taking advantage of the amenability of *Tribolium* for systemic and potent RNAi-mediated gene silencing [26], we knocked-down MMPs, TIMP, and RECK and observed phenotypes indicating MMP functions particularly during metamorphosis and embryogenic organogenesis of trachea and intestine. Furthermore, knock-down of MMP-1 resulted in animals significantly more susceptible to infection by the entomopathogenic fungus *Beauveria bassiana* than control (RNAi) animals providing evidence for a direct role of MMP-1 in insect innate immunity. This particular insect MMP is most related to mammalian MMP-19 whose functions in cutaneous immune response have recently been demonstrated [27]. Results provide evidence of the evolutionarily conserved and distinct roles of MMPs and their physiological inhibitors in both metazoan development and innate immunity.

## Results

### MMPs, TIMP, and RECK are evolutionarily conserved

**Figure 1:**
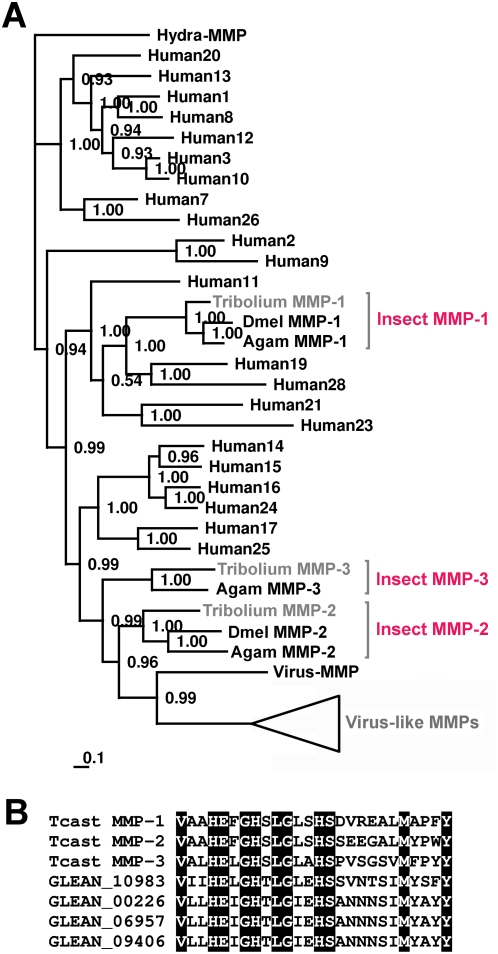
Phylogenetic analysis of *Tribolium* MMPs along with MMPs from other organisms. A Bayesian protein tree, with posterior probabilities, was generated using aligned MMP sequences from *T. castaneum*, *Anopheles gambiae*, *Drosophila melanogaster*, *Homo sapiens*, and *Heliothis zea virus 1*. A *Hydra vulgaris* MMP was used as outgroup. This analysis revealed that *Tribolium* MMP-1 is orthologous to other insect MMP-1s and *Tribolium* MMP-2 to MMP-2 from *Anopheles* and *Drosophila*. Interestingly, *Tribolium* MMP-3 clades with MMP-3 from *Anopheles*, but a MMP-3 orthologue is obviously absent in *Drosophila*. Insect MMP-1 proteins show highest relation to human MMP-19 and MMP-28 whereas the other insect MMPs group together with human membrane-anchored MMPs (MMP-14 to MMP-17, MMP-24 and MMP-25). In contrast to *Anopheles* and *Drosophila*, *Tribolium* has numerous genes that are most closely related to a MMP from *Heliothis zea virus 1*. The scale bar represents the substitutions per site according to the model of amino acid evolution applied. (B) Three *Tribolium* MMPs and virus-derived *Tribolium* MMP proteins have catalytic domains with conserved MMP specific active site sequences (MMP active site consensus sequence: HEXGHXXGXXHSX_6_M). For better overview sequences of only four virus-derived MMPs are shown.

BLAST searches of the complete *Tribolium* genome using human and *Drosophila* MMP sequences resulted in the identification of three Metazoa-like and ten probably virus-derived MMP genes. Phylogenetic analysis of *Tribolium* MMPs together with 22 MMPs from humans, two from *Drosophila melanogaster*, three from *Anopheles gambiae*, and one from the basal animal *Hydra vulgaris* revealed that *Tribolium* MMP-1 (XP_968822) is orthologous to MMP-1 from *Drosophila* and *Anopheles*, which are most closely-related to human MMP-19 and MMP-28 (Fig. 1A). *Tribolium* MMP-2 (XP_969495) is orthologous to MMP-2 from *Drosophila* and *Anopheles* and, interestingly, *Tribolium* MMP-3 (XP_972146) groups together with MMP-3 from *Anopheles* (Fig. 1A). A corresponding MMP-3 ortholog is absent in the *Drosophila* genome, suggesting its loss during fly evolution. Insect MMP-2 and insect MMP-3 clade with human membrane-anchored MMPs. Furthermore, we identified ten *Tribolium* specific MMP sequences (Glean 10983, Glean 00226, Glean 06957, Glean 09406, Glean 10302, Glean 03344, Glean 06957, Glean 01812, Glean 16133, and Glean 04281) that are most closely related to a MMP from the entomopathogenetic dsDNA virus *Heliothis zea virus 1* with yet unknown function. Interestingly, similar genes are also present in the genomes of other entomopathogenic viruses, e.g. *Trichoplusia ni ascovirus 2c* (YP_803380) and *Agrotis segetum granulovirus* (YP_006303), indicating a yet unknown but conserved role during virus infection of insects which may include host tissue histolysis. Some of the *Tribolium* virus-derived genes are located on a particular chromosomes such as Glean 10983 which is located on chromosome LG 9 and are expressed at the mRNA level since we amplified a corresponding cDNA by RT-PCR. In contrast, other predicted genes are located on contigs that are yet not attributed to a specific chromosome and need sequence verification. The deduced amino acid sequences of all *Tribolium* MMPs include the specific catalytic domains containing the conserved motif HEXGHXXGXXHSX_6_M, with the three histidine residues critical for catalytic activity by binding one zinc ion involved in proteolysis, the active site residue glutamic acid, and a conserved seven amino acid methionine-containing 1,4-β-turn named Met-turn [28] (Fig. 1B).

**Figure 2:**
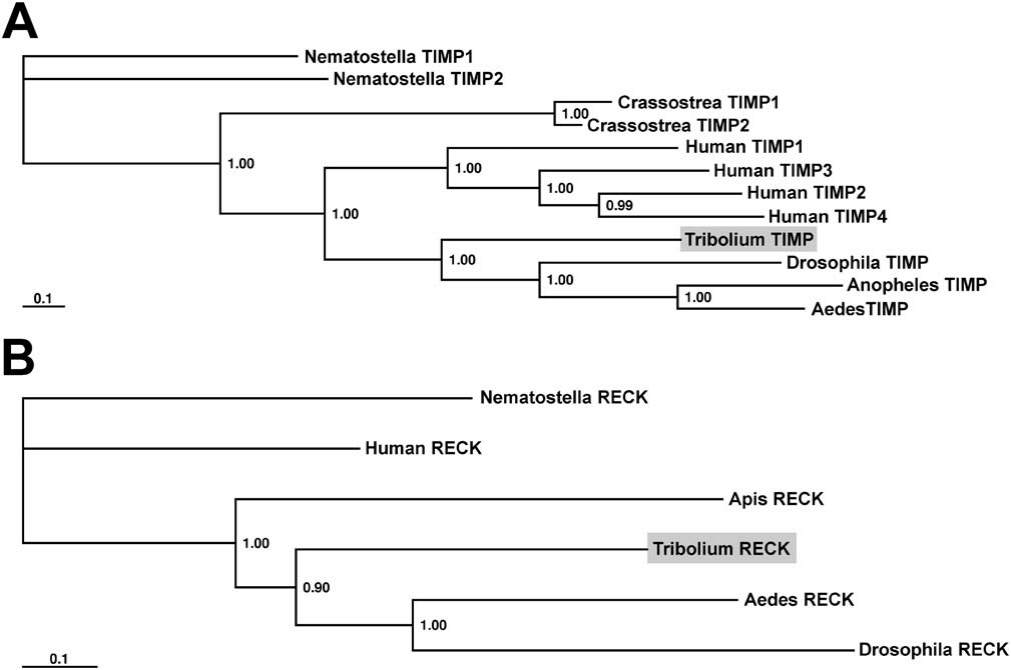
Phylogenetic analysis of *Tribolium* TIMP and RECK along with homologs from other organisms. (A) Aligned TIMP and (B) RECK protein sequences from *T. castaneum* along with homologs from other animals, were used to generate bayesian protein trees. *Tribolium* TIMP and RECK (indicated by gray shading) group well with other insect orthologs. Humans and some other animals have two or more TIMP homologs. Posterior probabilities are plotted at the nodes.

Furthermore, in the *Tribolium* genome, we identified one TIMP (Fig. 2A) and one RECK homolog (Fig. 2B) which share significant sequence similarities to counterparts from other animals including the basal sea anemone *Nematostella vectensis* and the complex Mammalia. Hence, MMPs, TIMP, and RECK are evolutionarily conserved ranging from most ancient to highly derived animals.

### Role of MMPs during metamorphosis: abnormal differentiation of antennae, compound eyes, appendages, and head by MMP-1 knock-down

**Figure 3:**
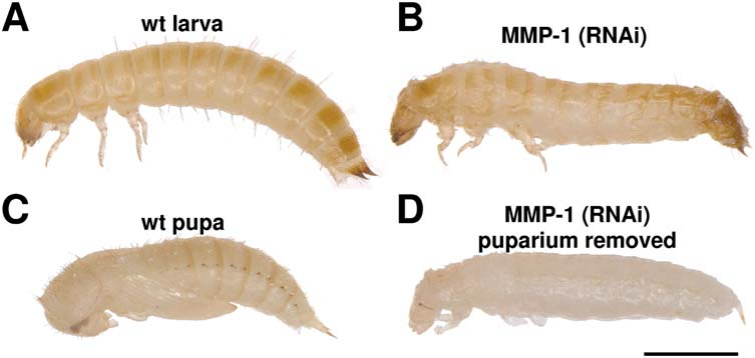
RNAi (MMP-1) knock-down *Tribolium* larvae arrest during larval to pupal transformation. Lateral views of whole bodies of wild-type larva (A) and arrested MMP-1 knock-down animals at the larval-pupal transformation (B) are shown. For comparison wild-type pupa (C) and MMP-1 knock-down animals that were removed from the puparium (D) are also shown. Scale bar, 1 mm.

To examine the functional roles of MMPs during *Tribolium* embryogenesis, larval growth, and metamorphosis, we injected dsRNA from *Tribolium* MMP-1,-2,-3, and MMP-4 (a virus-derived *Tribolium* MMP, Glean 10983), respectively, into larval, pupal, and adult stages and monitored their further development. Both efficiency and selectivity of each RNAi knock-down experiment were confirmed by RT-PCR analyses (data not shown). Last instar larvae or pupae injected with MMP-2, MMP-3, and MMP-4 dsRNA, respectively, showed no defects and developed to normal imagoes, like the control dsRNA treated animals. In contrast, knock-down of MMP-1 resulted in animals that arrest development and die during early metamorphosis (Fig. 3A,B). Though they showed a dorsal split in the thorax cuticula indicating initiation of ecdysis, the larvae were unable to shed their puparium. After removal of the apolysed larval cuticle the treated animals looked like pupal-larval intermediates with elongated body shape and untanned cuticle (Fig. 3C,D).

**Figure 4:**
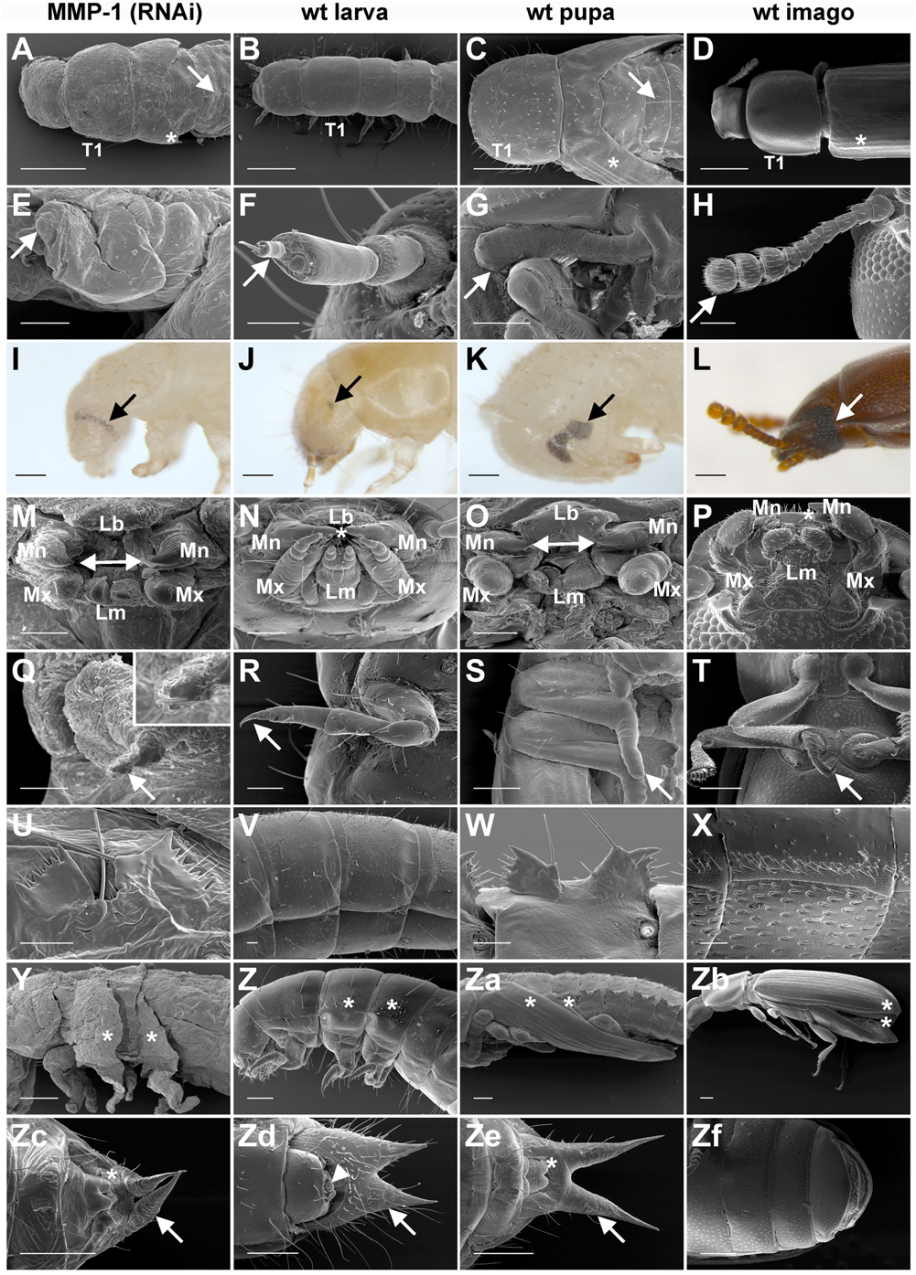
MMP-1 knock-down phenotype. MMP-1 knock-down animals were analyzed in detail in comparison to wild-type larva, pupa and imago. (A–D) MMP-1 knock-down animals had early pupa-like dorsal features including thorax segment 1 (T1), wings (asterisks) and a pupa-specific abdominal line of setae (arrows). (E–H) Their antennae were thick and un-segmented (tips of antennae are indicated by arrows). (I–L) MMP-1 (RNAi) larvae displayed premature compound eyes (pupal ommatidia) and exhibited reduced larval stemmata (arrows). (M–P) The mouth of MMP-1 (RNAi) phenotype animals exhibited a pupa-like morphology; mandible (Mn), maxilla (Mx), labrum (Lb), labium (Lm), gap between mandibles (indicated by arrow lines in pupa and MMP-1 knock-down phenotype), interlocking of mandibles (indicated by asterisks in larva and imago). (Q–T) MMP-1 (RNAi) larvae had shortened legs with a double inchoate claw similar to that observed in pupa (tip of legs are indicated by arrows). (U–X) Gin-traps were present in MMP-1 knock-down animals and pupae but were absent in wild-type larvae and imagoes. (Y–Zb) In MMP-1 (RNAi) animals wings were rudimentally developed (asterisks) (Zc–Zf), but genital papillae (asterisks, covered in adults) developed into those of pupae, replacing larval pygopods (arrow-head). Furthermore, urogomphi were elongated and pupa-like (arrows, not present in imago). Scale bars: A–D, 500 µm; E–H and M–T, 100 µm; I–L and Y–Zf, 200 µm; U–X, 50 µm.

Further examination revealed distinct visible abnormalities in some structures. The compound eye, for example, developed several rows of ommatidia but in abnormal arrangement when compared to wild-type pupae (Fig. 4I). The thorax of MMP-1 (RNAi) animals developed a pupa-like, not fully developed pronotum and the dorsal abdomen revealed a line of short setae, cuticular structures observed in the pupal stage (Fig. 4A,C). The wings were rudimentary (Fig. 4A,Y) but other characteristics like gin traps (Fig. 4U), genital papillae, and urogomphi were well developed (Fig. 4ZC). Mouthparts (Fig. 4M) were nearly fully developed including a pupa-like labrum and separated mandibles with a gap between the incisors (Fig. 4M). The antennae were thick (Fig. 4E), the head antrorsed (Fig. 4I), and the legs short and distant though they did possess the double claws that normally develop in pupae and are typical for the adult leg (Fig. 4Q).

When MMP-1 dsRNA was injected into pupae no distinct phenotype appeared and individuals developed to normal adults exhibiting normal egg production, suggesting no importance during the pupa to imago transition. Interestingly, the fourth instar larvae injected with MMP-1 dsRNA developed and molted normally into the final instar larval stage and reached the prepupal stage, suggesting no essential involvement of MMP-1 in ecdysis between larval stages. However, achieving the prepupal stage they then arrested and showed the same lethal phenotype as described above.

**Figure 5:**
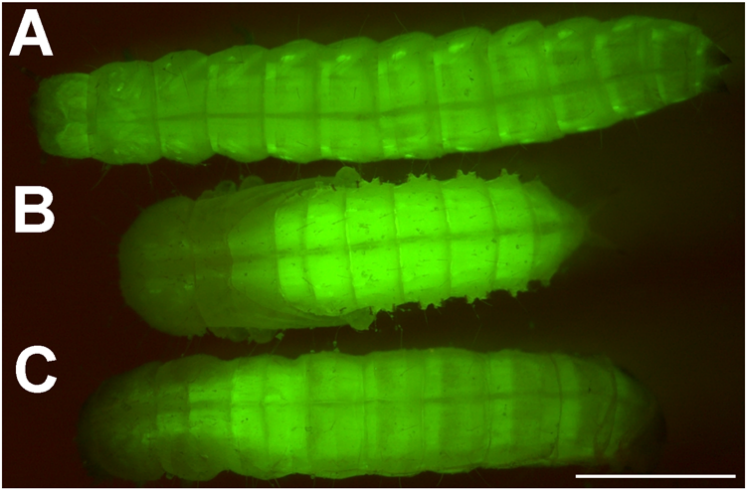
Muscle development in MMP-1 (RNAi) animals. (A) Pig-19 larva showed muscle specific EGFP-fluorescence throughout the whole body, with a notably strong expression at its lateral sides. (B) In control (RNAi) Pig-19 pupa the muscle EGFP-fluorescence was predominantly localized in the abdomen and visible as parallel strands. (C) MMP-1 (RNAi) larva showed a pupa-like localization of EGFP-fluorescence of muscles in the abdomen with additionally significant fluorescence in the thorax. Scale bar, 1 mm.

Using Pig-19 strain beetles, which express EGFP in muscle tissues [29] we examined muscle development. Control larvae exhibited well developed muscle fluorescence in the head and in the whole body (Fig. 5A) whereas in control pupae, fluorescence of muscles was more concentrated at the abdomen and organized in parallel strands (Fig. 5B). Observation of MMP-1 (RNAi) phenotype revealed that muscles had been mainly remodeled to pupal fate but without achieving final developmental stage (Fig. 5C).

**Figure 6:**
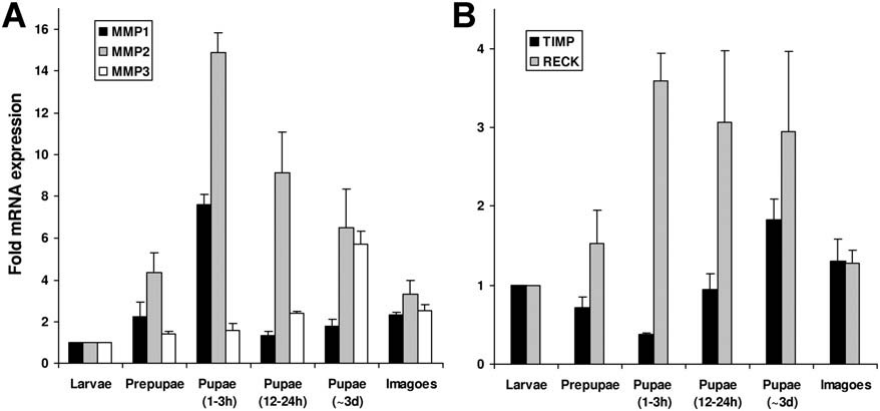
Quantitative real time RT-PCR analysis of transcriptional levels of *Tribolium* MMPs during metamorphosis. (A) The mRNA levels of MMP-1 (black bars), MMP-2 (gray bars), and MMP-3 (white bars) of different metamorphosis stages is shown relative to their expression levels in last instar larvae. Expression rates of MMP-1 increased 4-fold during prepupal stage and 8-fold during early pupal stage (1–3 h) and declined soon after this to basal levels. In contrast, MMP-2 mRNA levels were determined to be elevated at all analyzed prepupal and pupal stages with the highest elevation (15-fold) at the early pupal stage when compared to larval stage. Interestingly, MMP-3 expression levels were not induced during early stages but in a later stage of metamorphosis. In 3 day old pupae, we determined a 6-fold elevated transcript level. (B) TIMP gene expression was repressed 2-fold in early metamorphosis and induced in later stages, whereas RECK gene expression was induced during all tested stages of metamorphosis. Expression levels of MMP-4 (Glean 10983) and 18S rRNA genes were not significantly different at all analyzed stages (data not shown). Results represent mean values of three independent determinations±S.D. from ten animals per stage that were pooled for analysis.

To determine at which stages of pupal morphogenesis genes of MMPs, TIMP, and RECK are expressed we analyzed RNA from final instar larvae, prepupae (pharate pupae), pupae of different ages, and imagoes by quantitative real-time RT-PCR. Our analysis revealed enhanced mRNA levels of MMP-1 and MMP-2 in early pupae when compared with corresponding samples from last instar larvae, whereas MMP-3 showed an increased transcriptional rate in 3-day old pupae (Fig. 6A). Expression rates of MMP-1 increased 4-fold from larval to prepupal stage and 8-fold to early pupal stage, whereas MMP-2 mRNA levels were determined to be elevated during whole metamorphosis with highest values at early pupal stage. When compared with mRNA levels from last instar larvae, RECK was induced about 3-fold during all analyzed stages of metamorphosis, whereas TIMP was 2-fold repressed in early pupal stages similar as recently described in *Drosophila* [30]–[31] and increased up to 2-fold in later pupal stages (Fig. 6B). Expression levels of the virus-derived MMP-4 and 18S rRNA genes were not significantly different at all analyzed stages.

### Examination of potential regulation of TGF-β-like Decapentaplegic (Dpp) by MMP activity

Page-McCaw et al. [22] noted that *Drosophila* MMP-1 mutant phenotype resembles the phenotype derived by mutation of Decapentaplegic (Dpp) supporting the hypothesis that MMPs may modulate the morphogen gradient directly or indirectly by degrading extracellular matrix proteins. Dpp is a bone morphogenetic protein (BMP)-type ligand that belongs to the TGF-β family with essential role during insect development. The Dpp gradient is regulated by a number of modulators that either bind Dpp, cleave Dpp-binding proteins or compete with Dpp for receptor-binding sites [25].

**Figure 7:**
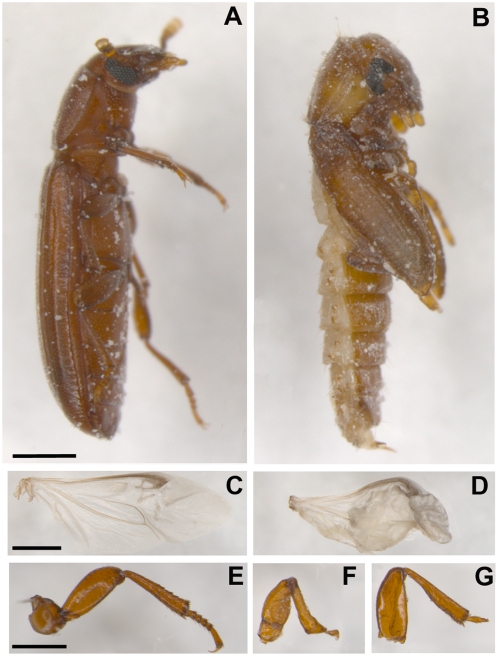
Phenotype arising upon Dpp knock-down in last instar larvae. (A) Lateral views of wild-type imago (A) and arrested dpp (RNAi) animal at pupal-adult transformation (B) are shown. This phenotype is dominant, however, some animals exhibited a milder phenotype, in which they ecdysed to imagoes but had affected wings (D) and leg deformations (F,G) of mainly the tarsus when compared to wild-type wing (C) and leg (E). Scale bar, A–B, 500 µm; C–G, 250 µm.

To investigate possible regulation of Dpp by MMP activity, we silenced Dpp expression in last instar *Tribolium* larvae and compared the resulting phenotype with that of MMP-1 (RNAi) phenotype. Recently, the essential role of Dpp during beetle embyrogenesis has been investigated [32] but to our knowledge this is the first study showing impact of Dpp on *Tribolium* metamorphosis. Efficiency of RNAi mediated Dpp knock-down was confirmed by RT-PCR analyses (data not shown). Interestingly, Dpp knock-down did not affect ecdysis between larval stages and larval to pupal transformation, but frequently resulted in an arrest of the pupal to adult transition leading to a phenotype with pupal morphology and with adult-like tanning of the cuticle (Fig. 7 A,B). Though this was the predominant phenotype, some animals exhibited a milder phenotype in which they ecdysed to imagoes but had affected inflated wings and leg deformations, mainly of the tarsus (Fig. 7 C–F), suggesting role in distal growth and differentiation of appendages. Since the *Tribolium* Dpp (RNAi) phenotype is strikingly different to MMP-1 (RNAi) phenotype our results indicate no or only little impact of Dpp on observed abnormalities of MMP-1 (RNAi) *Tribolium* beetles.

### Role of MMPs during embryonic development: abnormal tracheal growth by MMP-1 knock-down and gut development by MMP-2 knock-down

The offspring of imagoes that were injected with MMP-1 dsRNA hatched normally and showed no external defects. However, closer examinations revealed that they display abnormal tracheal development resulting in small breaks of lateral tracheal trunks between two or three segments at the posterior end of larva (data not shown). Despite these tracheal defects the animals showed no changes in their further development and emerged normally to imagoes.

**Figure 8:**
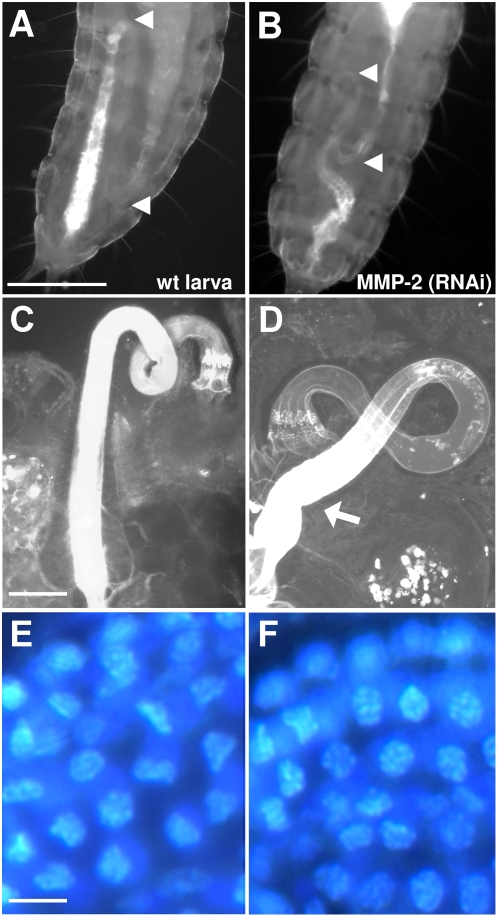
MMP-2 knock-down in adults result in offspring with gut defects. *Tribolium* larvae that are exposed to UV-light, resulting in auto-fluorescence of their cuticle and intestine. (A) Auto-fluorescence of the posterior end of a wild-type first instar larva showed an intestine that is normally s-shaped in a typical way in up to three segments (arrow-heads). (B) In contrast, examination of the posterior end of a first instar MMP-2 (RNAi) larva revealed an abnormal intestine with a twisted hind gut and a half truncated ileum loop. Closer examination of dissected hind guts from wild-type (C) and MMP-2 (RNAi) (D) larvae revealed no obvious differences except a kink at the end of the colon of MMP-2 (RNAi) larvae (indicated by an arrow). Gut epithelium seems not to be affected since cell appearance by DAPI-mediated nucleus staining indicated no differences. Scale bars, A–B, 100 µm; C–D, 50 µm; E–F, 5 µm.

Injection of dsRNA of MMP-2, MMP-3, and MMP-4, respectively, into last instar larvae or pupae had no effects on further development and morphology of imagoes. They exhibited a regular egg production and their offspring emerged at expected rates. While the offspring of MMP-3 (RNAi) or MMP-4 (RNAi) animals developed normally to adults, the first instar larvae of MMP-2 (RNAi) animals showed a distinct wandering behavior and died after few days. Closer examination revealed defects in their gut development. The hind gut of wild-type *Tribolium* is wound up in a typical way; the forward expanding ileum is transferred into the posterior turning colon and rectum (Fig. 8A). In contrast, the offspring of MMP-2 (RNAi) animals showed a deviant coiled hind gut; the turn of the ileum, which extends to three segments in control larvae, was half reduced and both the ileum and the colon were contorted and twisted with each other (Fig. 8B). Microscopic examination of dissected guts revealed no obvious differences between hind guts from MMP-2 (RNAi) and control (RNAi) larvae except a kink at the end of colon suggesting influence of MMP-2 on specific molecular guidance cues of gut organogenesis (Fig. 8CD).

### MMP activity and gene expression is induced in stimulated *Tribolium* hemocytes

**Figure 9:**
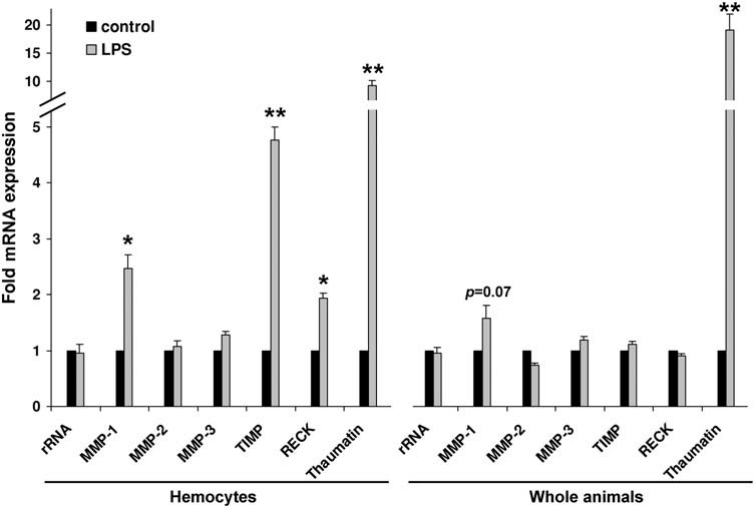
Quantitative real time RT-PCR analysis of transcriptional levels of *Tribolium* MMPs, TIMP, and RECK, along with immune-inducible thaumatin during innate immune responses. The mRNA levels of MMP-1, TIMP, and RECK were significantly induced 2 to 5-fold in hemocytes in response to immune-challenge along with the immune-inducible thaumatin gene. In contrast, in whole animal preparations containing mainly fat body tissue no significantly induced expression of tested genes could be observed except immune-inducible thaumatin, although a tendency towards MMP-1 induction was detected. 18S rRNA gene expression was not influenced by treatment, and expression levels of MMP-2 or MMP-3 were not significantly different upon immune-challenge. Results represent mean values of three independent determinations±S.D. from ten animals that were pooled for analysis. Statistically significant differences were determined using Student's t-test and are indicated (*, p<0.05; **, p<0.01).

**Figure 10:**
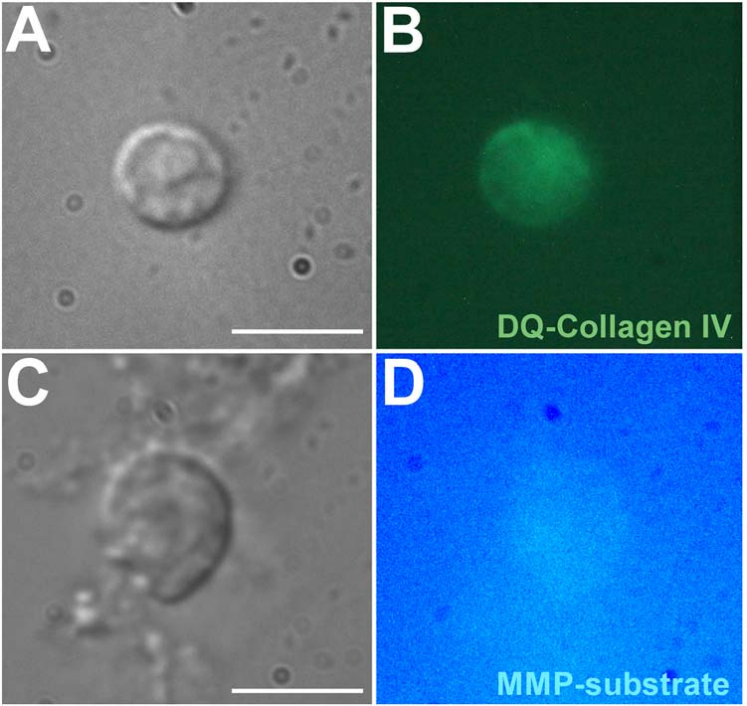
Detection of MMP-like collagenolytic activity on the surface of stimulated *Tribolium* hemocytes. Typical granular hemocytes from *Tribolium* (A,C) exhibited MMP-like enzymatic activity localized at their surface by hydrolyzing DQ™ collagen type IV resulting in green fluorescence (B) and of quenched, fluorogenic MMP-1/MMP-9 peptidic substrate resulting in bright blue fluorescence (D), respectively. Scale bar, 10 µm.

Insect hemocytes are highly motile macrophage-like cells fulfilling immune-related functions including phagocytosis, nodulation, and hemolymph coagulation [33]–[35]. Recently, we have identified a lepidopteran MMP which is induced in response to septic injury in immune-competent hemocytes [36]. Therefore, we investigated here the potential role of MMPs in insect innate immunity using model host insect *T. castaneum* [37]. Upon septic injury we observed significantly increased mRNA levels of about 2 to 5 fold of MMP-1, TIMP, and RECK, respectively, in immune-competent *Tribolium* hemocytes along with the immune-inducible antifungal *Tribolium* thaumatin (Fig. 9A). In contrast, mRNA levels of MMP-2 and MMP-3 were not significantly influenced by the treatment. In whole animals including the fat body, which is responsible for massive immune-induced production of antimicrobial peptides we determined only a weak and not significantly induced expression of MMP-1 (Fig. 9B). These results suggest that MMP-1 functions mainly at the cellular level of innate immunity and little in the systemic immune response. To confirm our results, we examined enzymatic activities of hemocytes and observed MMP-like enzymatic activity localized at the surface of stimulated hemocytes, which lead to the degradation of both fluorescently labeled collagen IV and MMP-1/MMP-9 peptidic substrate (Fig. 10A,B).

### Knock-down of MMP-1 significantly reduces survival time of infected beetles

**Figure 11:**
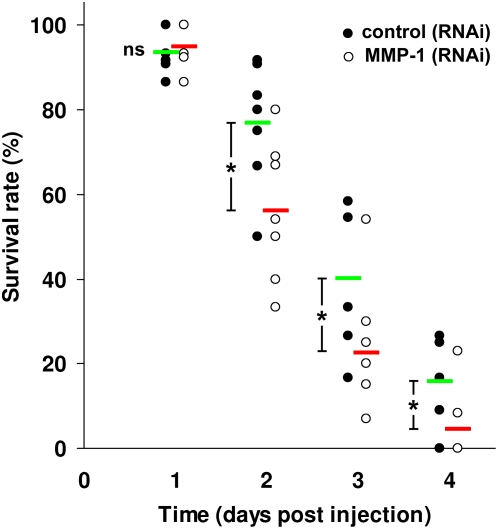
RNAi (MMP-1) knock-down *Tribolium* imagoes are more susceptible to infection by the fungal entomopathogen *B. bassiana*. Injection of *Beauveria bassiana* spores into the hemocoel of *Tribolium* imagoes resulted in killing within 4 to 5 days. MMP-1 knock-down animals died significantly faster than wild type animals. Values of seven independent trials including 10–15 animals per trial are shown for control (RNAi) animals and MMP-1 (RNAi) animals. Mean values of surviving control (RNAi) animals are shown in green and of surviving MMP-1 (RNAi) animals in red. Statistically significant differences were determined using Student's t-test and are indicated (*, p<0.05). Ns, not significant.

To obtain direct evidence that MMPs are involved in insect innate immunity, we knocked-down MMPs, TIMP, and RECK, respectively, in *T. castaneum* imagoes. Subsequently, three days later we infected them with the fungal entomopathogen *Beauveria bassiana* by injecting conidia spores into the hemocoel. Survival time of infected larvae was determined by counting live and died animals for several days upon infection. MMP-1 (RNAi) beetles died significantly faster than control RNAi animals, indicating an essential role of MMP-1 in proper insect innate immunity (Fig. 11). In contrast, knock-down of MMP-2, MMP-3, MMP-4, TIMP, and RECK, respectively, resulted in no altered susceptibility of beetles to *B. bassiana* (data not shown).

### Purification of recombinant *Tribolium* MMP-1 and determination of its collagenolytic activity

**Figure 12:**
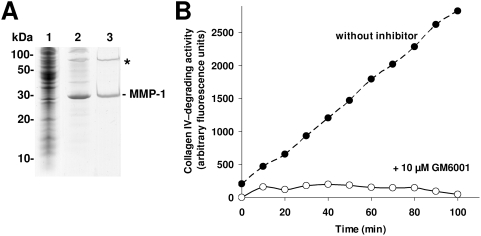
Purification and refolding of recombinant *Tribolium* MMP-1 and determination of its collagenolytic activity. (A) MMP-1 (lane 2) was purified to apparent homogeneity from the urea-soluble *E. coli* inclusion bodies fraction (lane 1) as observed by SDS–PAGE analysis. After refolding in appropriate buffer, we observed the MMP-1 protein band (according to the calculated molecular mass of about 30.3 kDa) and an additional band with an estimated two-fold molecular weight that may correspond to a dimer of MMP-1 (indicated by an asterisk). Molecular mass standards are indicated in kDa. (B) Collagen-IV degrading activity of recombinant *Tribolium* MMP-1 was monitored using DQ™ collagen (type IV from human placenta). The reaction was found to be linear for at least 100 min under examined conditions. This activity was abolished in the presence of 10 µM GM6001.

To investigate the enzymatic properties of MMP-1, we produced recombinant *Tribolium* MMP-1 in *E. coli. Tribolium* MMP-1 was purified from the urea-soluble *E. coli* inclusion bodies fraction by immobilized metal affinity chromatography (Fig. 12A). Auto-activation of purified and refolded protein resulted in active MMP-1 which was able to degrade collagen IV *in vitro* (Fig. 12B). Under examined conditions the reaction velocity of one µg recombinant *Tribolium* MMP-1 was 165±48 ng DQ™-collagen type IV per hour which was abolished in the presence of the specific MMP inhibitor GM6001 (Fig 12B). For comparison, using the same experimental conditions, we measured the reaction velocity of one µg recombinant human MMP-19 that was kindly provided by Radislav Sedlacek (Prague, Czech Republic) and found that it was in a similar range as that observed for recombinant *Tribolium* MMP-1 (data not shown).

## Discussion

Our results reveal that the *in vivo* functions of MMPs in the model beetle *T. castaneum* are wide ranging. Use of dsRNA-meditated gene silencing revealed that MMPs play essential roles during embryogenesis as well as metamorphosis. Furthermore, our results provide the first direct evidence that MMPs are involved in regulating innate immunity in insects.

In *Tribolium*, dsRNA-mediated genetic interference has been used recently to unravel novel functions of developmental transcription factors and morphogens in insect embryogenesis and metamorphosis [24]. Some of the regulative processes have been demonstrated to be similar, whereas others to be strikingly different to processes known from *Drosophila*. For example, in *Tribolium*, patterning of its dorsoventral axis is controlled by homologous genes that are also used in *Drosophila*, but rather than relying on maternal information, Dorsal and Dpp are part of two coordinated ancestral self-organized systems in the beetle embryo [32]. In contrast to *Drosophila*, *Tribolium* has an ancestral type of development, including a short germ band embryo with gradual segmentation and continuously limbs growth starting from an appendage bud shared with most insects and other arthropods as well as with vertebrates [25]. Hence, findings from *Tribolium*, when compared to findings from *Drosophila* and other animal model organisms, provide a deeper and a more comprehensive understanding of conserved as well as derived molecular mechanisms.

### Metamorphosis

Here, we show that RNAi-mediated silencing of MMP-1 leads to an arrest in the initial stage of *Tribolium* pupation resulting in phenotypes with altered development of antennae, compound eyes, wings, legs, and head. As a first step towards addressing molecular mechanisms behind this phenotype, we investigated the potential responsibility of the prominent morphogen Dpp because results from *D. melanogaster* suggested an possible role of Dpp on observed MMP-1 (RNAi) phenotype. Knock-down of Dpp leads to an arrest of pupal to adult transition during adult morphogenesis giving a phenotype with pupal characteristics and adult-like tanning of the cuticula. Since MMP-1 (RNAi) animals arrest in an earlier stage of metamorphosis, we propose that Dpp has no or only little impact on MMP-1 knock-down phenotype.

In *Drosophila*, MMPs have been co-opted for maggot specific metamorphosis processes including head evasion and notum connation [38]. Both processes have recently been demonstrated to depend on imaginal disc eversions which require basement membrane remodeling by particularly MMPs [39]. Since *Tribolium* has no imaginal discs and instead limbs grow continuously starting from an appendage bud, we postulate that MMP-1 (RNAi) phenotypes of *Tribolium* may also be a result of altered basement membrane remodeling thereby leading to abnormal pupal tissue development and differentiation. Moreover, beside basement membrane remodeling other molecular processes such as specific cell migration and autophagic cell death processes, which are known to be regulated by MMPs in mammals, may be also involved in observed phenotypes and will be investigated in future studies. Confirming this assumption, it has been recently demonstrated in *Drosophila* salivary glands that the ecdysone/EcR/USP complex induces primary response gene expression during metamorphosis, including the E74, BR-C, and E93 transcription factor genes and that these genes induce secondary-response genes, including apoptosis regulators and MMP-1 while simultaneously repressing TIMP expression [30].

Collagen degradation has been attributed to MMPs since they were first identified in 1962 by Gross and Lapière in tadpole tails during metamorphosis [40]. Interestingly, we found that *Tribolium* harbors genes encoding at least five different collagen isoforms (XP_970497, XP_970652, XP_001811664, XP_971465, and XP_974458), whereas *Drosophila* have only two collagen isoforms [41], indicating that the *Tribolium* extracellular matrix has a much more complex composition. We found that recombinant and purified *Tribolium* MMP-1 is able to degrade collagens, with enzymatic activity that was in a similar range to that observed for human MMP-19. Moreover, we found that several insect species including *Tribolium* have three MMP paralogs, whereas *Drosophila* lacks one of these MMPs, insect MMP-3. The existence of a third MMP homolog in *Tribolium* and in other insects may directly correlate with the need for a sophistically regulated degradation of a more complex extracellular matrix. However, we found no obvious change in phenotype during metamorphosis when knocking-down MMP-2 and MMP-3. This is quite surprising giving the fact that MMP-2 and MMP-3 are highly up-regulated during metamorphosis, and *Drosophila* MMP-2 mutants are severally affected during the larval-pupal transition. We postulate that MMP-2 and MMP-3 have overlapping functions during metamorphosis since they are more related to each other than to MMP-1. Furthermore, one or more virus-derived MMPs may have been co-opted in *Tribolium* for larval tissue histolysis during metamorphosis.

### Embryogenesis

An interesting finding is that the gut development of *Tribolium* embryos is affected by MMP-2 knock-down. This is in striking contrast to findings from *Drosophila* whose MMPs are not essentially involved in embryogenesis [22]. Since *Drosophila* has lost one MMP during evolution we propose that regulation of gut development by MMP-2 may reflect a more ancestral state and further investigation of role of MMPs in other insects such as *Anopheles* or *Apis*, may help to unravel this role in more detail.

The tracheal defects caused by MMP-1 (RNAi) are not detrimental for development and survival of *Tribolium* larvae. This is in striking contrast to findings from the fruit fly since *Drosophila* MMP-1 mutants die by hypoxy resulting by affected tracheal trunks. A possible explanation is that the more ancient tracheal system of beetles, which harbor functional spiracles ( = stigmata) at all body segments helps to survive tracheal trunk breaks, whereas *Drosophila* larvae which are particularly adapted to their ecological niche and have therefore reduced most spiracles [42] except anterior and posterior ones die by suffocation.

### Innate immunity

Besides developmental functions, we provide evidence that at least MMP-1 exerts pleitrophic functions by additionally mediating proper innate immune defense against pathogens because MMP-1 (RNAi) beetles were more susceptible to the entomopathogenic fungus *B. bassiana*. We further determined elevated levels of MMP activity as well as gene expression of MMP-1, TIMP, and RECK in stimulated immune-competent hemocytes. This is in agreement with recent observations that activated human immune cells express high levels of MMPs [43] and that immune-competent hemocytes from the mollusk *Crassostrea virginica* exhibit increased levels of MMP activities [44] as well as induced gene expression of TIMP [45], in response to immune-challenge. In insect hemocytes, MMPs may be required for tissue transmigration to reach the site of infection or to activate cytokines or antimicrobial peptides, similar to what has been recently shown for mammalian lymphocytes [1]–[6], whereas TIMP and RECK may have regulatory functions. It is also noteworthy, that we have recently described the ability of the insect innate immune system to sense collagen IV fragments that represent endogenous danger signals during infection [46], [47]. Accruing collagen IV fragments by increasing hemocyte-derived MMP activity, therefore, may serve as a potentiating factor of innate immune responses against pathogens according to the proposed danger model by Matzinger [48].

Since insect MMP-1s are most closely related to mammalian MMP-19 and MMP-28, we postulate that some functions may have been conserved between insects and mammals. Indeed, it has recently been shown that MMP-28 expression is induced during dermal wound healing [49] and that MMP-19 has indispensable functions in cutaneous homeostasis by modulating epidermal proliferation [50], [51], cutaneous immune responses [27] as well as skin tumorigenesis [52], [53]. Furthermore, human MMP-19 associates with the surface of human monocytes and macrophages [54] and impacts the maturation and response of mammalian T-cells that are involved in both innate and adaptive immunity [27]. Thus, insect model organisms lacking adaptive immune system may help to elucidate direct role of MMPs in innate specific immune responses. Moreover, we have recently determined septic injury-inducible MMP in the planarian *Schmidtea mediterranea* [55] belonging to the Lophotrochozoa, suggesting that MMPs exhibit evolutionarily conserved functions in innate immunity of many diverse animals. In sum, results from *Tribolium* MMPs shed new light on MMP roles in insect development and immunity in particular and on evolutionarily conserved *in vivo* roles of MMPs in Metaozoa in general.

## Materials and Methods

### Animals, RNAi and infection experiments

Wild-type *Tribolium castaneum* strain San Bernardino and strain Pig-19 expressing EGFP in muscle tissues [29] beetles, kindly provided by Gregor Bucher (Georg August University, Goettingen), were reared on whole-grain flour with 5% yeast powder at 32°C in darkness as described previously [56]. Ambion MEGAscript T7 kit (Applied Biosystems, Austin, TX) was used to prepare dsRNA according to the manufacturers' protocol. Appropriate templates for *Tribolium* MMP-1 (XP_968822), *Tribolium* MMP-2 (XP_969495), *Tribolium* MMP-3 (XP_972146), a virus-derived MMP (MMP-4, Glean 10983), *Tribolium* TIMP (XP_001810132), and *Tribolium* RECK (XP_974450) were generated by PCR using gene specific primers containing a T7 polymerase promoter sequence at their 5′ end (Table 1) and purified with the Macherey&Nagel (Germany) NucleoSpin Extract II Kit prior use. Injection of dsRNA into animals was performed with a micromanipulator under a dissecting stereomicroscope. Animals were anaesthetized for 5 min on ice before they were affixed to double-stick tape. About 0.2 µg dsRNA was injected dorsolaterally into the second/third abdominal segment of beetles. Controls received the same volume of dsRNA of the lepidopteran *Galleria mellonella* insect metalloproteinase inhibitor gene (AY330624) [57], which is not present in *T. castaneum*. Infection experiments with the entomopathogenic fungus *Beauveria bassiana* (Boverol®, Fytovita, Ostrožská Lhota, Czech Republic) were performed by injecting 10^4^ conidia spores into the hemocoel of imagoes, and survival rate was determined every 24 hours at 25°C. For septic injury experiments we injected 10^4^ heat-killed BL21(DE3) *Escherichia coli* cells (Invitrogen, Carlsbad, CA) into the hemocoel.

**Table 1:** Primer sequences used in the present study.

Gene	Forward primer 5′–3′	Reverse primer 5′–3′
*Quantitative real time RT-PCR primer*		
RT-18S rRNA	ATGGTTGCAAAGCTGAAACT	TCCCGTGTTGAGTCAAATTA
RT-MMP-1	CGATAGGGCCTTTGCTGTGG	CACCCCCTGGACTCACTCGT
RT-MMP-2	GCGATGTGTGTGTGCTGTGG	CGTTGCACCGAATCATCGAC
RT-MMP-3	GTGGGTTGGACGGGAATGAG	GGCGCCTCCTCCGCTATATT
RT-MMP-4	CGTTGTGGATTGCCTGATGC	CACATGTGCACGCCACGTTA
RT-TIMP	GGCAGTAACGGTGGCGTACC	TCACGAAGTCGGCTCTGCAA
RT-RECK	ACAGCTGCCGAAAAGCTTGC	GGCGTTCTAAGCAGCGGAAA
RT-Thaumatin	GGCAACGGGGTTATTGCTTG	ACGTGTCAGGTGTGCCGAAA
*Primers used for RNAi experiments*		
RNAi-MMP-1	TAATACGACTCACTATAGGGAGATCCAGAGTTTTGCCGGTCTC	TAATACGACTCACTATAGGGAGATTGGAAGTCCGAATCGTCGT
RNAi-MMP-2	TAATACGACTCACTATAGGGAGACGGGATTTGATGGGAATGGA	TAATACGACTCACTATAGGGAGATTTGTCGTCGTCGGGGAGTT
RNAi-MMP-3	TAATACGACTCACTATAGGGAGACTCCGAAGGGTGGGGAAAAC	TAATACGACTCACTATAGGGAGACGTCAAAGGAGGCGTCACAA
RNAi-MMP-4	TAATACGACTCACTATAGGGAGACGTTGTGGATTGCCTGATGCT	TAATACGACTCACTATAGGGAGAGGGATTTTTGCGTTGTTGTTGTTG
RNAi-TIMP	TAATACGACTCACTATAGGGAGATGACCGCATCTGACGAAGCA	TAATACGACTCACTATAGGGAGA
		TCCGAACCACCGAGAAATGG
RNAi-RECK	TAATACGACTCACTATAGGGAGAATTGCCACAACCTGCCCAAA	TAATACGACTCACTATAGGGAGA
		TCGTTTGCACCGTCACGAAA
RNAi-Dpp	TAATACGACTCACTATAGGGAGATCGACACTGTTGCCCCTTTT	TAATACGACTCACTATAGGGAGATGGTTGGTTTTGGGGTCTTG
RNAi-IMPI (control)	TAATACGACTCACTATAGGGAGACGGTGGAGCCTGCGATAATG	TAATACGACTCACTATAGGGAGACGACGGTGGAGGGGAGTCAA

### Sequence alignments and phylogenetic analyses

Sequences were aligned using the blosum62 program [58]. Accession numbers of MMPs used for sequence alignment and phylogenetic analyses are as follows: *Tribolium castaneum* (Tribolium MMP-1, XP_968822; Tribolium MMP-2, XP_969495; Tribolium MMP-3, XP_972146; virus-derived MMPs: Glean 10983, Glean 00226, Glean 06957, Glean 09406, Glean 10302, Glean 03344, Glean 06957, Glean 01812, Glean 16133, and Glean 04281), *Anopheles gambiae* (Agam MMP-1, XP_001688107; Agam MMP-2, XP_320653; Agam MMP-3, XP_554330), *Drosophila melanogaster* (Dmel MMP-1, NP_523852; Dmel MMP-2, NP_995788), *Homo sapiens* (Human19, Q99542; Human28, Q9H239; Human11, P24347; Human21, Q8N119; Human23, O75900; Human17, Q9ULZ9; Human25, Q9NPA2; Human14, P50281; Human15, P51511; Human16, P51512; Human24, Q9Y5R2; Human20, O60882; Human12, P39900; Human13, P45452; Human1, P03956; Human8, P22894; Human3, P08254; Human10, P09238; Human7, P09237; Human26, Q9NRE1; Human2, P08253; Human9, P14780), *Heliothis zea virus 1* (AAN04364), and *Hydra vulgaris* MMP (AAD45804). Accession numbers of TIMPs used for sequence alignment and phylogenetic analyses are as follows: *Tribolium* (XP_001810132), *Nematostella vectensis* (TIMP1, XP_001625449; TIMP2, XP_001625448), *Drosophila melanogaster* (NP_524301), *Anopheles gambiae* (XP_001231186); *Aedes aegypti* (EAT34208); *Crassostrea gigas* (TIMP1, AAG42824; TIMP2, AAT73610); *Homo sapiens* (TIMP1, P01033; TIMP2, P16035; TIMP3, P35625; TIMP4, Q99727). Accession numbers of RECKs used for sequence alignment and phylogenetic analyses are as follows: *Tribolium* (XP_974450), *Nematostella vectensis* (XP_001635685), *Drosophila melanogaster* (NP_648733), *Apis mellifera* (XP_392031); *Aedes aegypti* (XP_001657759); *Homo sapiens* (NP_066934). For phylogenetic reconstruction, we used the software package MrBayes 3.1.2 [59], which combines Bayesian inference and Markov chain Monte Carlo convergence acceleration techniques known as Metropolis coupling. The best fixed-rate model of amino acid evolution was determined by model jumping among nine possible models. The model with the overall highest posterior probability was WAG [60] for MMPs, TIMPs, and RECKs. The average standard deviation of split frequencies at 10^6^ generations was 0.0021 for MMPs, and 0.001 and 0.0018 for TIMP for RECK, respectively, at 10^5^ generations. These values indicated that the two chains that were run in each analysis converged on similar results. The 50% majority rule tree presented here was constructed from all sampled trees with the first 25% of all trees ignored as burn in. Posterior probabilities plotted at the nodes can be interpreted as the probability that the tree or clade is correct [61].

### Quantitative real time RT-PCR

RNA was isolated using TriReagent isolation reagent (Molecular Research Centre, Cincinnati, Ohio, USA) according to the instructions of the manufacturer and quantitative real time RT-PCR were performed with the real-time PCR system Mx3000P (Stratagene, La Jolla, California, USA) using the FullVelocity SYBR® Green QRT-PCR Master Mix (Stratagene), as described previously [56]. We used appropriate primers (Table 1) along with 0.1 ng of RNA per reaction to amplify 18S rRNA and 10 ng of RNA per reaction to amplify selected *Tribolium* genes. Primers were selected using the primer3 software [62] and were purchased from Sigma (Taufkirchen, Germany).

### Imaging and scanning electron microscopy

EGFP expression was observed using a fluorescence stereomicroscope (Leica MZ 16F) equipped with a GFP Plus filter set (excitation filter: 480/40 nm, barrier filter: 510 nm). Images of cuticle and gut autofluorescence were obtained on a microscope (Zeiss Axioplan 2) using a fluorescence optic. For electron microscopy animals were fixed in 80% ethanol and dehydrated in a graded series of ethanol, critical point dried using CO_2_ in a Balzers CPD 030, mounted on aluminium stubs, gold coated using a sputter coater 0712 B (Balzer/Leitz), and photographed with a Philips XL 20 scanning electron microscope.

### Expression and purification of recombinant *Tribolium* MMP-1

A cDNA fragment of *Tribolium* MMP-1, encoding the pro- and catalytic domains, was amplified by PCR with oligonucleotides TC-MMP1-for-EX2 -3′-ATGGCGCCCAGTGGAAGCTCT-5′ and TC-MMP1-rev-EX3 -3′-CTTCCCGTACAAAGCCTGGAT-5′. The resulting PCR amplification product was subsequently cloned by use of the TA Expression Kit into the pCRT7/CT-TOPO expression vector (Invitrogen). This construct allowed the production of *Tribolium* MMP-1 in *Escherichia coli* with a carboxy-terminal histidine tag. BL21(DE3) *E. coli* cells (Invitrogen, Carlsbad, CA) were used for production of recombinant *Tribolium* MMP-1. Optimal protein production was observed when cultures were grown at 32°C without IPTG to an optical density of 5–6 at 600 nm. The cells were harvested by centrifugation and cell pellets were stored at −20°C until use. We purified the recombinant protein under denaturating conditions using 8 M urea and 100 mM sodium phosphate buffer at pH 8 since all *Tribolium* MMP-1 protein was found in insoluble inclusion bodies. All purification steps were monitored by SDS-PAGE analysis using 15%-Tris–tricine gels. The bacterial cell pellet was resuspended in saline buffer and disintegrated by french press. After centrifugation of the cell lysate, proteins from inclusion bodies were solubilized in 8 M urea and 100 mM sodium phosphate buffer, pH 8. After a subsequent centrifugation step *Tribolium* MMP-1 was purified from the supernatant using immobilized metal affinity chromatography on Protino® Ni-IDA resin (Macherey Nagel, Düren, Germany) according to the instructions of the manufacturer. The protein was desalted, refolded, and concentrated using Amicon® Ultra-4 centrifugal filter devices (Millipore, Billerica, MA). In brief, the protein solution was concentrated by ultrafiltration to a volume of about 100 µl and diluted in 5 mM NaH_2_PO_4_, pH 7, to a volume of 4 ml. This procedure was repeated five times. For over night auto-activation, recombinant MMP was incubated in reaction buffer (50 mM Tris-HCl, 150 mM NaCl, 5 mM CaCl_2_, 1 mM 2-mercaptoethanol, 10 µM ZnCl_2_, pH 7) for 16 h at 25°C. Protein concentrations were measured using the method of Bradford with bovine serum albumin as a standard. The concentration of purified *Tribolium* MMP-1 was determined in 20 mM sodium phosphate buffer (pH 6.5) containing 6 M guanidium hydrochloride using UV spectroscopy (ε_280_ = 34,380 mM^−1^ cm^−1^).

### Collagen IV degrading activity assay

Collagen IV degrading activity of recombinant *Tribolium* MMP-1 was monitored using 100 µg/ml DQ™ collagen (type IV from human placenta, fluorescein conjugate; Invitrogen) in reaction buffer (50 mM Tris-HCl, 150 mM NaCl, 5 mM CaCl_2_, 10 µM ZnCl_2_, pH 7) at 30°C, according to the recommendations of the manufacturer. Fluorescence was measured using the real-time PCR system Mx3000P (Stratagene, La Jolla, CA, USA) as a fluorescence reader with filter settings for excitation at 492 nm and emission at 516 nm. The broad-spectrum MMP inhibitor GM6001 (Merck KGaA, Darmstadt, Germany) was used to inhibit MMP activity.

### *Ex vivo* microscopic analysis of *Tribolium* hemocytes

Larvae were punctured with a sterile needle, and obtained hemolymph samples were dropped on microscope slides and immediately mixed with buffer (NaH_2_PO_4_, pH 7) including no substrate, 100 µg/ml DQ™ collagen (type IV from human placenta, fluorescein conjugate; Invitrogen, Carlsbad, CA), and 500 µM fluorogenic MMP-1/MMP-9 substrate (Calbiochem, Merck KGaA, Darmstadt, Germany), respectively, and were monitored with an Axioplan 2 microscope (Zeiss, Jena, Germany) with a fluorescence filter according to manufacturers' recommendations.
